# Intimate partner violence: a cross-sectional study in women treated in the Brazilian Public Health System

**DOI:** 10.31744/einstein_journal/2021AO6584

**Published:** 2021-11-10

**Authors:** Kennya Formiga, Victor Zaia, Maria Vertamatti, Caio Parente Barbosa

**Affiliations:** 1 Centro Universitário FMABC Santo André SP Brazil Centro Universitário FMABC, Santo André, SP, Brazil.

**Keywords:** Violence against women, Domestic violence, Exposure to violence, Gender-based violence, Unified Health System

## Abstract

**Objective:**

To determine the prevalence and types of violence suffered by women and to identify the gender attitudes related to the situation.

**Methods:**

This was a descritive, cross-sectional study incluiding 343 women who were assisted at the Brazilian Public Health System in countryside city in northeastern of Brazil. All participants were volunteers and they invited to participate during consultation at a Basic Health Unit. As participants, they filled out the World Health Organization Violence Against Women Questionnaire and responded to a sociodemographic questionnaire.

**Results:**

The victims were, on average, 20.3 years old, and 53.2% of them were married. There was a prevalence of 52.9% of psychological violence, 30.5% of physical violence, and 12.3% of sexual violence. Participants reported alcoholism (67%) and jealousy (60.8%) as triggers to violence. The main psychological abuses were insults and humiliation. In terms of physical violence, the major ones were pushes and slaps. The sexual violence most reportedwere sexual intercourse against the will of the woman and sexual intercourse because of fear of the partner. A portion of the participants justified violence due to women’s infidelity, refusal to have sex, and disobedience to her husband.

**Conclusion:**

Education in gender equality as a measure of opposition to the culture of female subjugation can reflect on the resignification of the violence suffered by them, and not on blaming the victim of violence by an intimate partner.

## INTRODUCTION

The World Health Organization (WHO) considers violence against women a public health problem, with consequences for families and economy.^(1)^ Among the forms of violence, the intimate partner violence (IPV) is one of them that has an impact on human rights, which is commonly committed by men against their partners.^(2,3)^

The United Nations (UN) defines violence against women as any act of gender-based violence that results in physical, sexual, or psychological harm or suffering in any form, including threats, coercion, arbitrariness, and deprivation of liberty. It is estimated that one of every three women (30%) worldwide have suffered some form of violence.^(4)^

Intimate partner violence tends to lead women victims into isolation, decrease their productivity and family income, as well as cause damage to their mental and reproductive health, enhance their well-being, and dignity. This generates losses for society and may have a negative impact on the education of new generations.^(5)^

In developing countries, data on violence suffered by women are scarce, either due to underreporting or to the violent behavior of the partner be not understood as such by the victim, or by the society. For this reason, studies are warranted to identify and report violence.^(5)^ In the absence of data that allow an intervention on the IPV situation, women often manage alone the trauma of violence, and avoid to expose their relationship, an behavior that perpetuates the anonymity about such cases.^(2)^

The United Nations General Assembly issued a document that characterizes violence against women,^(4)^ in an attempt to improve the production of data on violence to establish more easily different public policies to prevent and eradicate such violence in the world.^(6)^ Among these policies there are the Convention of Belém do Pará, promoted by the Inter-American Commission on Human Rights, which indicates possible actions to punish and eradicate violence against women.^(7)^ In addition, the law #11.340 issued by the Brazilian government, namely Maria da Penha’s law that intensifies the rigor of punishments against violence suffered by women,^(8)^ and provide more support to Brazilian institutions to guarantee health and prevent violence.^(9)^

Among these Brazilian institutions is the Brazilian Public Health System (*Sistema Único de Saúde*) has an important role to receive and treat women victims of IPV. Primary Health Care (PHC), the front door of the Brazilian Public Health System, provides the first care for violence victims, in addition, PHC is responsible to provide the necessary treatments for them to recover from the violence suffered.^(10)^

## OBJECTIVE

To determine the prevalence and types of violence suffered by women and to identify gender attitude towards intimate partner violence.

## METHODS

This was a descritive, cross-sectional study on IPV and gender attitudes. The Strengthening the Reporting of Observational Studies in Epidemiology (STROBE) protocol was used.^(11)^

The study was developed by the *Centro Universitário FMABC* at a Basic Health Unit located in the city of Cajazeiras (PB), Northeast Region of Brazil. At the time of this study, the Human Development Index (HDI) of the region was 0.679.^(12)^ Data collection occurred between January 12 and December 20, 2018 in a specific office designated by the Basic Health Unit to ensure confidentiality of participants.

The sample size was calculated using the G*Power 3.1.9.4 program, considering the statistical tests used, and sample power of 90%, with p≤0.05. The minimum sample size indicated was 320 participants. However, a total of 350 women were invited, as an attempt to preview possible non-adherence to the study. Of women invited to the study, seven refused to participate because they were unavailable to talk about the topic.

The inclusion criteria were to be a woman aged 18 or older and be alone at time of the interview. Women who were unable to participate and to respond autonomously to the survey were excluded. All participants were volunteers and they were invited to join the study at the Basic Health Unit where they were seeking medical care.

### Instruments

We used the Brazilian validate version of the World Health Organization Violence Against Women (WHO-VAW) to access the variables that were object of the study (intimate partner violence and gender attitudes).^(3,13)^ This is a specific questionnaire for violence indicated by the WHO that allows control over data collection, which include 12 independent sections that address various topics related to IPV. The instrument used includes a Likert type scale with two or three degrees (participants responded to certain claims as “agree”, “disagree” and “don’t know”, or only “agree” and “disagree”, when the statement referred to their partner). Sections of the questionnaire used were concerned women’s attitudes toward gender roles, the daily life of the interviewee and her intimate partner, characterization of the injuries suffered, and the impact and coping strategies used by women to face the situation of violence.

For general characterization of participants, a sociodemographic questionnaire was used. This questionnaire included questions on marital status (single, married, separated, divorced or widowed), age (in years), whether they experienced episodes of violence (yes or no), number of violence episodes (one, two and three or more episodes), whether they lived with the aggressor (yes or no), and what type of violence they had suffered (psychological, sexual and physical).

### Ethical aspects

The study was approved by the Research Ethics Committee of the *Centro Universitário FMABC* (CAAE: 11784013.7.0000.0082, report # 420.206). An informed verbal consent form was signed that guaranteed the confidentiality of all information collected. At no time during the study, participants were identified, and an ID number was assigned to each participant. The signature of the consent form was waived to guarantee the anonymity of participants.

### Procedures and data analysis

A single researcher contacted the participants, she was trained not to express verbal and physical reactions to the participants’ answers, and to follow the standardized questions in the instruments to the interview. These measures were adopted to reduce biases during data collection. Answers were recorded in a research notebook using codes and without identifying respondents.

Subsequently, all responses were transcribed into the (SPSS) program by two independent researchers. Possible transcription errors were verified and corrected. The missing values in data were checked and maintained because we considered that some participants decided not to answer some questions due to personal reasons. The valid percentage was adopted to the analyses in cases of categorical variables. Mean and standard deviations were used for continuous variables. For comparisons between categorical variables, the χ^2^ test was used by following the numerical premise of the test.^(14)^ A 5% significance value was adopted in all statistical tests.

## RESULTS

The sample was composed of 343 women with mean age of 20.3 (±8.82) years. Of participants, 77.5% reported living with their aggressor. Of participants, 39.6% of the reported to had suffered three or more episodes of psychological violence in last year, 42.0% reported physical violence at the same period, and 31.8% sexual violence within the last year (Table 1).

**Table 1:** Demographic characteristics

Characteristics				n (%)*
Marital status				
	Married			183 (53.4)
	Divorced			9 (2.6)
	Separated			29 (8.5)
	Single			122 (35.6)
Currently living with the aggressor				
	Yes			265 (77.5)
	No			77 (22.5)
Have you experienced any violence in your life				
	Yes			343 (100)
	No			0 (0)
Type and number of violence episodes in the last year				
	Psychological			
		1		67 (35.8)
		2		46 (24.6)
		≥3		74 (39.6)
	Physical			
		1		32 (29.9)
		2		30 (28.0)
		≥3		45 (42.0)
	Sexual			
		1		18 (40.9)
		2		12 (27.3)
		≥3		14 (31.8)
Age of participants, mean±SD				20.3±8.82

* Variations in total may occur because of lack of responses.

Table 2 indicates gender attitudes included in the WHO-VAW. Among participants, 42.9% indicated that “a wife should obey her husband, even if she disagrees with him”; 36.7% considered that “if a woman is mistreated, no one outside the family should intervene”. In 30% of the cases, the participant mentioned that “the husband prevents her from seeing friends”, and 42.3% reported that “their husband gets angry if they talk to other men”.

**Table 2:** Affirmation regarding gender relationship

Affirmation		Agree n (%)	Disagree n (%)	Do not know n (%)
A good wife obeys her husband even if she disagrees with him*		147 (42.9)	186 (54.2)	10 (2.9)
It is important for a man to show his wife who is in charge*		119 (34.7)	219 (63.8)	5 (1.5)
A woman should choose her own friends even when her husband does not agree*		151 (44.0)	179 (52.2)	13 (3.8)
A wife should have sex with her husband even if she doesn’t want to have sex*		36 (10.5)	300 (87.5)	7 (2.0)
If a man mistreats his wife, others outside the family should not intervene*		214 (62.4)	126 (36.7)	3 (0.9)
Your spouse†		Yes	No	
	Prevents you from seeing your friends	103 (30.0)	240 (70.0)	
	Restricts your contact with your family	43 (15.5)	290 (84.5)	
	Insists on always knowing where you are	133 (38.8)	210 (61.2)	
	Ignores you or treats you with indifference	85 (24.8)	258 (75.2)	
	Becomes angry if you talk to another man	148 (42.3)	195 (55.7)	
	Constantly accuses you of being unfaithful	53 (15.5)	290 (84.5)	
	Must allow you to seek health care for yourself	29 (8.5)	314 (89.7)	

* Affirmation with three degrees of Likert type response

† affirmation with two degrees of Likert type response.

We observed that 52.9% of the participants suffered psychological violence, 30.5% physical violence, and 12.3% sexual violence (the same woman could indicate more than one type of insult suffered, which generated an absolute total greater than the number of participants). In terms of type of insult, 42.2% reported having suffered verbal insults and 25.9% to be pushed, in addition to 9.3% who reported to be forced into degrading sexual practices (Table 3).

**Table 3:** Type of insult due to violence suffered

Type of insult		No* n (%)	Yes* n (%)
Psychologic			
	Insult	188 (54.8)	155 (42.2)
	Humiliation	251 (73.2)	92 (26.8)
	Intimidation	240 (70.0)	103 (30.0)
	Threat	273 (79.6)	70 (20.4)
Physical			
	Slapping	262 (76.4)	81 (23.6)
	Pushing	254 (74.1)	89 (25.9)
	Punching	302 (86.3)	41 (11.7)
	Kicking	316 (92.1)	27 (7.7)
	Strangulation	329 (95.9)	14 (4.1)
	Threat	312 (91.0)	31 (9.0)
Sexual			
	To force sexual intercourse	311 (90.7)	32 (9.3)
	To have sexual intercourse for fear	314 (91.5)	29 (8.5)
	Degrading sexual practice	321 (93.6)	22 (6.4)

* Total is higher than the total of women who suffered violence from their partners, because it considered the amount of types of insults experienced by them.

Alcohol abuse (67%) and jealousy (60.8%) were indicated as possible triggers of violence. Although 36.5% of the participants reported that there were not clearly reason for an acting of violence, some of them considered that there were some possible justifications for a woman to be beat by her husband. Of participants, 19.4% believe that the justification was infidelity; for 8.5% suspicion of infidelity; for 3.2% disobedience to her husband; for 2.9% refusal to have sexual intercourse; for 2.3%, unsatisfactory housework, and for 2.0% if she asks if her husband had another woman.

The highest frequency of violence episodes occurred proportionally among divorced and married women. Women who did not live with their aggressor showed higher frequency of psychological violence than those who leave with them (p=0.01), however, these women also reported lower frequency of physical (p<0.001) and sexual (p=0.01) violence. Women whose partners were literate had lower proportions in the frequency of psychological (p=0.042) and physical (p=0.031) violence than those whose partners were illiterate. Higher education of the partner was proportionally related to lower frequency of psychological (p=0.021), physical (p=0.004) and sexual (p=0.040) violence. When women were literate, lower frequencies of episodes of physical (p=0.003) and sexual (p=0.023) violence were found. Higher education of the woman was related to lower frequency of episodes of psychological violence (p=0.005) (Table 4).

**Table 4:** Relationship of violence with sociodemographic characteristics

Variable		Violence					
		Psychological		Physical		Sexual	
		No	Yes	No	Yes	No	Yes
Marital status, n (%)							
	Married	83 (45.4)	100 (54.6)	132 (72.1)	1 (27.9)	161 (88.0)	22 (12.0)
	Divorced	1 (11.1)	8 (88.9)	4 (44.4)	5 (55.6)	6 (66.7)	3 (33.3)
	Separated	8 (27.6)	21 (72.4)	13 (44.8)	16 (55.2)	20 (69.0)	9 (31.0)
	Single	65 (53.3)	57 (46.7)	88 (72.1)	34 (27.9)	113 (92.6)	9 (7.4)
	p value (χ^2^)*	0.01		<0.001		0.01	
Live with the aggressor, n (%)							
	Yes	133 (50.2)	132 (49.8)	193 (72.8)	72 (27.2)	236 (89.1)	29 (10.9)
	No	21 (29.2)	51 (70.8)	41 (56.9)	31 (43.1)	59 (81.9)	13 (18.1)
	p value (χ^2^)*	<0.001		<0.001		0.10	
Literate partner, n (%)							
	Yes	151 (47.6)	166 (52.4)	223 (70.3)	94 (29.7)	278 (87.7)	39 (12.3)
	No	7 (26.9)	19 (73.1)	13 (50.0)	13 (50.0)	22 (84.6)	4 (15.4)
	p value (χ^2^)*	0.042		0.031		0.587	
Formal education of the partners, years, n (%)							
	8	59 (40.4)	87 (59.6)	90 (61.6)	56 (38.4)	123 (84.2)	23 (15.8)
	11	70 (51.1)	67 (48.9)	104 (75.9)	33 (24.1)	121 (88.3)	16 (11.7)
	5	22 (64.7)	12 (35.3)	29 (85.3)	5 (14.7)	34 (100.0)	0 (0.0)
	p value (χ^2^)*	0.021		0.004		0.040	
Are you literate?, n (%)							
	Yes	158 (46.6)	181 (53.4)	236 (69.6)	103 (30.4)	298 (87.9)	41 (12.1)
	No	0 (0.0)	4 (100.0)	0 (0.0)	4 (100.0)	2 (50.0)	2 (50.0)
	p value (χ^2^)*	0.063		0.003		0.023	
Participant’s formal education, years, n (%)							
	8 years	44 (35.2)	81 (64.8)	78 (62.4)	47 (37.6)	104 (83.2)	21 (16.8)
	11 years	79 (52.7)	71 (47.3)	108 (72.0)	42 (28.0)	134 (89.3)	16 (10.7)
	15 years	35 (54.7)	29 (45.3)	50 (78.1)	14 (21.9)	60 (93.8)	4 (6.2)
	p value (χ^2^)*	0.005		0.059		0.084	

* test χ^2^.

## DISCUSSION

Intimate partner violence is the behavior that causes physical, sexual or psychological harm to a woman, which is caused by current or former intimate partners.^(3)^ Based on results of this study we observed that level of formal the education of woman and her partner were protective factors to prevent the occurrence of violence, especially when both partners had a higher level of formal education.^(15-19)^

The findings showed a higher frequency of psychological violence in separated and divorced women, indicating that stability in a relationship could favor a lower occurrence of this type of event.^(6,20)^

All participants in this study indicated that they had suffered some type of IPV. A possible explanation for this occurrence could be a conversion of factors such as the sum of different participants who had experienced different types of violence, cultural issues of submission of the feminine to the masculine,^(3)^ and given the research site that were a Basic Health Unit that is part of Brazilian Public Health System.^(10)^

The highest prevalent type of violence was psychological, which confirmed the initial hypothesis of this study. This type of violence was followed by physical and sexual, which are similar results of those reported in the literature.^(17,20,21)^ Although psychological violence does not leave physical marks, it has equal negative and harmful effects to women’s health.^(5,22)^

Among the types of psychological violence, insult and intimidation are understood as the forms of violence that cause the most emotional damage and lower self-esteem, since they intend to control actions, behaviors, beliefs and decision-making.^(8,9,23)^

The frequency of occurrence of physical violence (30%) in this study remained consistent with the rates reported by other countries, such as Japan (13%) and Peru (61%). The violent behavior of partners could be associated with the feeling of domination and control over the partner,^(24)^ which tends to justify the finding on the recurrence of the episode of violence.

The identified sexual violence (12.3%) in our study is higher than in studies conducted in Nepal^(24)^ and Mexico,^(2)^ but it is lower than in a study with Thai women.^(25)^ Sexual violence rarely occurs isolated, there is also risk factor for several health problems among women, with lasting repercussions in their lives.^(3)^

Among the most frequent types of sexual violence, forced sexual intercourse was identified. Other studies^(3,6)^ have indicated the occurrence of sexual intercourse to try to maintain their own integrity. This situation could be explained by the discrepancy in the understanding of what is sexual violence to women and to men, since there is still the social idea that women should perform the sexual act only by the obligation of satisfying men’s desire.^(26)^

When participants were asked on what they considered predisposing factors to the act of IPV, most of them indicated the abuse of alcohol by the partner. Other studies^(16,24)^ have emphasized that alcohol should not be used by power authorities as an explanation for violent behavior, but as an aggravating factor of it. Furthermore, some studies^(2,3,16)^ indicated that the habit of alcohol abuse can increase the risk of sexual violence up to four times, physical violence by ten times, and psychological violence by five times.

Concerning gender attitudes, it was possible to identify that, although there was no plausible reason that justifies an act of violence,^(3)^ some of the participants considered possible explanations to justify the violence suffered, such as female infidelity and disobedience. Similar findings were found in a study by the WHO study carried out in urban areas of Brazil, Japan, Namibia, Serbia and Montenegro.^(3)^ The same pattern was also observed in Bangladesh, Ethiopia, Peru, Samoa, Egypt^(1)^ and Thailand.^(25)^

The consideration of a possible justification to the violence suffered denotes attitudes of submission and conformism of the woman in relation to her partner, which confirmed the second hypothesis of this study, since continuous submission over the years, decreases self-esteem and the ability to think and react.^(27)^ For this reason, the hope of ending of the situation of violence gives way to conformism.^(3,28)^

Conformism tends to trivialize violence, which can be seen as something natural. This causes some women to assume passive postures, as if this were the most appropriate response to a possible change in the behavior of the partner.^(27)^

Attitudes of submission and subservience, such as those described here, may be associated with a rigid and traditional education based on subordination to the male gender. The declaration 6 of the Inter-American Convention on the Prevention, Punishment and Eradication of Violence Against Women seeks to deconstruct, by stating that every woman has the right not to be discriminated against and to be valuable and educated, to ensure equality between genders.^(7)^

The present study has limitations, such as having been carried out at a single place and include a convenient sample. These facts may generate results with limitations in their generalizability. Furthermore, the division of the population into smaller groups, such as by type of violence, gender attitude, or family income resulted of small sub-samples that prevented to further explore the data using statistical analyses.

Despite limitations, this study was able to present the experience of different types of violence reported by participants. In addition, we could identify occurrences and characteristics that allow better management of the studied population as an attempt to reduce the IPV suffered by them.

Finally, our findings indicated the need to promote education for gender equality and higher self-esteem among women. These may help to inhibit the practice of IPV and help to establish interventions to control violence against everyone involved, not only to victims.

## CONCLUSION

This study described the physical, psychological and sexual violence committed by the intimate partner against women who received care assistance at the Brazilian Public Health System in a countryside city in northeastern of Brazil. The highest prevalence of violence was psychological, followed by physical and sexual violence. Level of formation education of women and their partners proved to be a protective factor against violence. Alcohol abuse was recognized as a predisposing factor for violent acts committed by the partner. The gender-related issue that raised attention was the identification of women who considered that there were explanations that justified the violence they have suffered, an issue that reflect gender culture experienced by the participants.
