# Supplementary material for: Intimate partner violence: a cross-sectional study in women treated in the Brazilian Public Health System
Source: Einstein (Sao Paulo). 2021 Nov 10;19:eAO6584. doi: 10.31744/einstein_journal/2021AO6584 (PMC8687701; doi:10.31744/einstein_journal/2021AO6584)
Supplement: Translation

# Translation: Violência cometida pelo parceiro íntimo: estudo observacional com mulheres atendidas no Sistema Único de Saúde

**Authors:** Kennya Formiga, Victor Zaia, Maria Vertamatti, Caio Parente Barbosa

**Affiliations:** 1 Santo André SP Brasil Centro Universitário FMABC, Santo André, SP, Brasil.

**Keywords:** Violência contra a mulher, Violência doméstica, Exposição à violência, Violência de gênero, Sistema Único de Saúde

## Abstract

**Objetivo:**

Verificar a prevalência dos tipos de violência sofridos por mulheres e identificar as atitudes de gênero em relação a eles.

**Métodos:**

Estudo descritivo, observacional com a participação de 343 mulheres atendidas no Sistema Único de Saúde em uma cidade no nordeste brasileiro. Todas as participantes eram voluntárias e estavam presentes em uma Unidade Básica de Saúde para realizar algum atendimento médico. As participantes preencheram o questionário *World Health Organization Violence Against Women* e responderam a um questionário sociodemográfico.

**Resultados:**

As vítimas tinham, em média, 20,3 anos de idade, e 53,2% eram casadas. Houve prevalência de 52,9% de violência psicológica, 30,5% de violência física e 12,3% de violência sexual. As participantes indicaram como disparadores de violência a bebida (67%) e o ciúme (60,8%). Os principais abusos psicológicos foram insultos e humilhação; os físicos foram empurrões e tapas, e os sexuais foram relação sexual contra a vontade da mulher e por medo do parceiro. Uma parcela das participantes justificou a violência sofrida com a infidelidade da própria mulher, a recusa em manter relações sexuais e a desobediência ao marido.

**Conclusão:**

A educação em igualdade de gênero como medida de contraposição à cultura de subjugo feminino pode refletir na ressignificação da violência sofrida e na não culpabilização da própria vítima de violência pelo parceiro íntimo.

## INTRODUÇÃO

A Organização Mundial da Saúde (OMS) considera a violência contra a mulher como um problema de saúde pública, com repercussão familiar e econômica.^(1)^ Dentre as formas de violência está a violência entre parceiros íntimos (VPI), com impacto sobre os direitos humanos, a qual é comumente praticada pelo homem contra sua parceira.^(2,3)^

A Organização das Nações Unidas (ONU) define a violência contra a mulher como a prática de qualquer ato de violência de gênero que resulte em danos físicos, sexuais, psicológicos, ou em qualquer forma de sofrimento, incluindo ameaças, coerção, arbitrariedade e privação da liberdade. Estima-se que uma em cada três mulheres (30%) no mundo sofra alguma forma de violência.^(4)^

A VPI tende a levar as mulheres vítimas ao isolamento, diminuindo sua produtividade e seus vencimentos, bem como provocando danos à saúde mental e reprodutiva e agravo do bem-estar e da dignidade. Isso gera prejuízos para a sociedade, com repercussões negativas para a educação de novas gerações.^(5)^

Nos países em desenvolvimento, os dados da violência sofrida pelas mulheres são escassos, ou por subnotificação, ou porque o comportamento violento do parceiro não é compreendido como tal pela vítima e nem pela sociedade. Justificam-se, assim, estudos contínuos para a identificação e o relato de violência.^(5)^ Na ausência de dados que permitam uma intervenção sobre a situação de VPI, a mulher tende a gerenciar os traumas da violência sozinha, evitando expor sua relação e perpetuando o anonimato sobre tais casos.^(2)^

No intuito de melhorar a produção de dados sobre a violência, a Assembleia Geral da ONU promulgou um documento que caracteriza a violência contra a mulher,^(4)^ para mais facilmente estabelecer diferentes políticas públicas que visem erradicar e prevenir tal violência no mundo.^(6)^ Dentre tais políticas, estão a Convenção de Belém do Pará, promovida pela Comissão Interamericana de Direitos Humanos, a qual indica ações possíveis para punir e erradicar a violência contra a mulher,^(7)^ e a lei 11.340, promulgada pelo governo brasileiro e conhecida como Lei Maria da Penha, que intensifica o rigor nas punições contra agressões sofridas pelas mulheres,^(8)^ dando maior corpo às instituições brasileiras, para garantir a saúde e a preservação da vítima de violência.^(9)^

Dentre essas instituições brasileiras, está o Sistema Único de Saúde (SUS), que é importante no acolhimento e no tratamento das mulheres vítimas de VPI. A Atenção Primária, uma das frentes do SUS, faz o primeiro atendimento dessas vítimas, além de ser a responsável pelos tratamentos necessários para que elas se recuperem da violência sofrida.^(10)^

## OBJETIVO

Verificar a prevalência dos tipos de violência sofridos por mulheres e identificar as atitudes de gênero em relação a eles.

## MÉTODOS

Estudo descritivo, observacional sobre violência cometida pelo parceiro íntimo e atitudes de gênero. Utilizou-se o protocolo de divulgação científica *Strengthening the Reporting of Observational Studies in Epidemiology* (STROBE).^(11)^

O estudo foi desenvolvido pelo Centro Universitário FMABC em uma Unidade Básica de Saúde localizada na cidade de Cajazeiras (PB), Região Nordeste do Brasil. O Índice de Desenvolvimento Humano (IDH) da região era de 0,679.^(12)^ A coleta de dados ocorreu entre 12 de janeiro e 20 de dezembro de 2018 em um consultório específico designado pela Unidade Básica de Saúde, para garantir o sigilo e a discrição às participantes do estudo.

O tamanho amostral foi calculado por meio do programa G*Power 3.1.9.4, considerando os testes estatísticos utilizados e poder amostral de 90%, com p≤0,05. A amostra mínima indicada foi de 320 participantes. Optou-se por convidar 350 mulheres, considerando possíveis perdas amostrais. Das mulheres convidadas a participar, sete recusaram-se, indicando não possuir disponibilidade para falar sobre o assunto.

Os critérios de inclusão das participantes foram ser mulher, com idade igual ou superior a 18 anos e o parceiro estar ausente no momento do convite/participação. Foram excluídas mulheres com incapacidade de participar e responder de forma autônoma à pesquisa.

Todas as participantes foram voluntárias e estavam presentes na Unidade Básica de Saúde, para realizar algum atendimento médico, tendo sido convidadas a participar do estudo.

### Instrumentos

Para o acesso às variáveis que foram objeto de estudo (violência pelo parceiro íntimo e atitudes de gênero), foi utilizado o *World Health Organization Violence Against Women* (WHO-VAW) validado para o Brasil,^(3,13)^ que é um questionário específico para violência indicado pela OMS e permite um controle sobre a coleta de dados, diferenciando-os em 12 seções independentes que abordam diversos temas relacionados à VPI. As respostas são do tipo Likert, com dois ou três graus (a participante respondeu a determinadas afirmações cujas possibilidades de resposta eram “concordo”, “discordo” e “não sei”, ou somente “concordo” e “discordo”, quando a afirmação se referiu ao parceiro). Foram utilizadas as seções do questionário que diziam respeito às atitudes das mulheres com relação aos papéis de gênero; ao cotidiano da entrevistada e de seu parceiro íntimo; à caracterização das lesões sofridas e ao impacto e enfrentamento pela mulher da situação de violência.

Para caracterização geral das participantes, utilizou-se um questionário sociodemográfico, com questões sobre estado civil (solteira, casada, separada, divorciada ou viúva), idade (medida em anos), se vivenciou episódios de violência (sim ou não), quantos episódios de violência (um, dois e três ou mais episódios), se convivia com o agressor (sim ou não) e qual tipo da violência sofrida (psicológica, sexual e física).

### Aspectos éticos

O estudo foi aprovado pelo Comitê de Ética em Pesquisa (CEP) do Centro Universitário FMABC (CAAE: 11784013.7.0000.0082, parecer 420.206). Contou-se com termo de consentimento verbal, no qual ficou assegurado sigilo sobre todas as informações coletadas. Em nenhum momento da pesquisa, as participantes foram identificadas, tendo sido atribuído um número de participação a cada uma. A assinatura do Termo de Consentimento foi dispensada pelo CEP, visando maior garantia de anonimato das participantes.

### Procedimento e análise dos dados

Uma única pesquisadora realizou o contato com as participantes, sendo treinada para não expressar reações verbais e físicas pelas respostas das participantes, além de se restringir às questões padronizadas pelos instrumentos utilizados. Tais medidas visaram à redução de vieses de coleta. As respostas eram anotadas em uma caderneta de pesquisa de maneira codificadas e sem identificação da respondente.

Em seguida, todas as respostas foram transcritas para o programa (SPSS) por dois pesquisadores independentes. Verificaram-se possíveis erros de transcrição, os quais foram corrigidos. Os valores ausentes nos dados foram checados e mantidos, por se compreender que algumas participantes optaram por não responder a alguma determinada questão, por motivo pessoal. Para as análises nos casos de variáveis categóricas, optou-se pelo percentual válido. Para as variáveis contínuas, usaram-se a média e o desvio-padrão. Para a comparação entre variáveis categóricas, utilizou-se o teste χ^2^ , sendo respeitada a premissa numérica do teste.^(14)^ Adotou-se o valor de significância de 5% em todos os testes estatísticos.

## RESULTADOS

A amostra foi composta de 343 mulheres, com média de idade de 20,3 (±8,82) anos. Indicaram conviver com o agressor 77,5% das participantes. Sobre a quantidade de episódios de violência sofridos no último ano, 39,6% das participantes relataram terem passados por três ou mais episódios de violência psicológica, 42,0% de violência física e 31,8% de violência sexual (Tabela 1).

**Tabela 1:** Características demográficas

Características				n (%)*
Estado civil				
	Casada			183 (53,4)
	Divorciada			9 (2,6)
	Separada			29 (8,5)
	Solteira			122 (35,6)
Convive com o agressor				
	Sim			265 (77,5)
	Não			77 (22,5)
Sofreu alguma violência ao longo da vida				
	Sim			343 (100)
	Não			0 (0)
Tipo e número de episódios de violência no último ano				
	Psicológica			
		1		67 (35,8)
		2		46 (24,6)
		≥3		74 (39,6)
	Física			
		1		32 (29,9)
		2		30 (28,0)
		≥3		45 (42,0)
	Sexual			
		1		18 (40,9)
		2		12 (27,3)
		≥3		14 (31,8)
Idade da participante, média±DP				20,3±8,82

* Variações na totalidade podem ocorrer devido a respostas ausentes.

A tabela 2 indica atitudes de gênero contidas no WHO-VAW. Dentre as participantes, 42,9% indicaram que “uma esposa deveria obedecer ao marido, ainda que discorde dele”; 36,7% consideraram que “se uma mulher for maltratada, ninguém de fora da família deveria intervir”. Em 30% dos casos, a participante referiu que “o esposo evita que ela veja amigos” e 42,3% que “ficam zangados se elas conversam com outros homens”.

**Tabela 2:** Afirmações sobre relação de gênero

Afirmação		Concordo n (%)	Discordo n (%)	Não sei n (%)
Uma boa esposa obedece ao marido mesmo que discorde dele*		147 (42,9)	186 (54,2)	10 (2,9)
É importante o homem mostrar à sua esposa quem é que manda*		119 (34,7)	219 (63,8)	5 (1,5)
Uma mulher deve escolher seus próprios amigos mesmo quando seu marido não concorda*		151 (44,0)	179 (52,2)	13 (3,8)
É obrigação da esposa manter relações sexuais com seu marido, mesmo sem ter vontade*		36 (10,5)	300 (87,5)	7 (2,0)
Se um homem maltrata sua esposa, outras pessoas de fora da família deveriam intervir*		214 (62,4)	126 (36,7)	3 (0,9)
Teu esposo†		Sim	Não	
	Evita que você veja seus amigos	103 (30,0)	240 (70,0)	
	Restringe seu contato com sua família	43 (15,5)	290 (84,5)	
	Insiste em saber sempre onde você está	133 (38,8)	210 (61,2)	
	Ignora-a ou a trata com indiferença	85 (24,8)	258 (75,2)	
	Fica zangado se você conversa com outro homem	148 (42,3)	195 (55,7)	
	Suspeita constantemente que você é infiel	53 (15,5)	290 (84,5)	
	Deve permitir que você procure um serviço de saúde para você mesma	29 (8,5)	314 (89,7)	

* Afirmações com três graus de resposta tipo Likert

† afirmações com dois graus de resposta tipo Likert.

Verificou-se que 52,9% das participantes sofreram violência psicológica, 30,5% física e 12,3% sexual (foi possível para uma mesma mulher ter indicado mais de um tipo de insulto sofrido, o que originou um total absoluto maior do que o número de participantes). Com relação ao tipo de insulto, 42,2% referiram ter sofrido insultos verbais e 25,9% empurrão, além de 9,3% terem sido forçadas à prática sexual degradante (Tabela 3).

**Tabela 3:** Tipo de insulto por violência sofrida

Tipo de insulto		Não* n (%)	Sim* n (%)
Psicológico			
	Insulto	188 (54,8)	155 (42,2)
	Humilhação	251 (73,2)	92 (26,8)
	Intimidação	240 (70,0)	103 (30,0)
	Ameaça	273 (79,6)	70 (20,4)
Físico			
	Tapa	262 (76,4)	81 (23,6)
	Empurrão	254 (74,1)	89 (25,9)
	Soco	302 (86,3)	41 (11,7)
	Chute	316 (92,1)	27 (7,7)
	Estrangulamento	329 (95,9)	14 (4,1)
	Ameaça	312 (91,0)	31 (9,0)
Sexual			
	Forçar ter relação sexual	311 (90,7)	32 (9,3)
	Relação sexual por medo dele	314 (91,5)	29 (8,5)
	Prática sexual degradante	321 (93,6)	22 (6,4)

* O total é superior ao total de mulheres que sofreram violência do parceiro, pois foi considerada a somatória dos tipos de insultos vivenciados por elas.

Foram indicados como possíveis desencadeadores da violência o abuso de álcool (67%) e o ciúme (60,8%). Ainda que 36,5% das participantes tenham descrito não existir motivo plausível para um ato de violência, algumas consideraram haver possíveis justificativas para que uma mulher apanhasse do esposo. Para 19,4% a justificativa era a infidelidade; para 8,5%, a suspeita de infidelidade; para 3,2%, a desobediência do esposo; para 2,9%, a recusa em manter relação sexual; para 2,3%, o trabalho doméstico insatisfatório, e, para 2,0%, se ela “perguntar se marido possui outra mulher”.

A maior frequência de episódios de violência ocorreu proporcionalmente com mulheres divorciadas e casadas. As mulheres que não conviviam com o agressor apresentaram maior frequência de violência psicológica do que as que conviviam (p=0,01), mas elas também relataram menor frequência das violências física (p<0,001) e sexual (p=0,01). As mulheres cujos parceiros sabiam ler e escrever apresentaram menores proporções na frequência das violências psicológica (p=0,042) e física (p=0,031) do que aquelas cujos parceiros não sabiam ler e escrever. Maior escolaridade do parceiro esteve proporcionalmente relacionada à menor frequência de violências psicológica (p=0,021), física (p=0,004) e sexual (p=0,040). Quando as mulheres sabiam ler e escrever, foram verificadas menores frequências de episódios de violência física (p=0,003) e sexual (p=0,023). Maior escolaridade da mulher se relacionou a menor frequência de episódios de violência psicológica (p=0,005) (Tabela 4).

**Tabela 4:** Relação da violência com características sociodemográficas

Variável		Violência					
		Psicológica		Física		Sexual	
		Não	Sim	Não	Sim	Não	Sim
Estado civil, n (%)							
	Casada	83 (45,4)	100 (54,6)	132 (72,1)	1 (27,9)	161 (88,0)	22 (12,0)
	Divorciada	1 (11,1)	8 (88,9)	4 (44,4)	5 (55,6)	6 (66,7)	3 (33,3)
	Separada	8 (27,6)	21 (72,4)	13 (44,8)	16 (55,2)	20 (69,0)	9 (31,0)
	Solteira	65 (53,3)	57 (46,7)	88 (72,1)	34 (27,9)	113 (92,6)	9 (7,4)
	Valor de p (χ^2^)*	0,01		<0,001		0,01	
Convivência com o agressor, n (%)							
	Sim	133 (50,2)	132 (49,8)	193 (72,8)	72 (27,2)	236 (89,1)	29 (10,9)
	Não	21 (29,2)	51 (70,8)	41 (56,9)	31 (43,1)	59 (81,9)	13 (18,1)
	Valor de p (χ^2^)*	<0,001		<0,001		0,10	
Parceiro sabe ler ou escrever, n (%)							
	Sim	151 (47,6)	166 (52,4)	223 (70,3)	94 (29,7)	278 (87,7)	39 (12,3)
	Não	7 (26,9)	19 (73,1)	13 (50,0)	13 (50,0)	22 (84,6)	4 (15,4)
	Valor de p (χ^2^)*	0,042		0,031		0,587	
Escolaridade parceiro, anos, n (%)							
	8	59 (40,4)	87 (59,6)	90 (61,6)	56 (38,4)	123 (84,2)	23 (15,8)
	11	70 (51,1)	67 (48,9)	104 (75,9)	33 (24,1)	121 (88,3)	16 (11,7)
	5	22 (64,7)	12 (35,3)	29 (85,3)	5 (14,7)	34 (100,0)	0 (0,0)
	Valor de p (χ^2^)*	0,021		0,004		0,040	
Você sabe ler ou escrever?, n (%)							
	Sim	158 (46,6)	181 (53,4)	236 (69,6)	103 (30,4)	298 (87,9)	41 (12,1)
	Não	0 (0,0)	4 (100,0)	0 (0,0)	4 (100,0)	2 (50,0)	2 (50,0)
	Valor de p (χ^2^)*	0,063		0,003		0,023	
Escolaridade da participante, anos, n (%)							
	8 anos	44 (35,2)	81 (64,8)	78 (62,4)	47 (37,6)	104 (83,2)	21 (16,8)
	11anos	79 (52,7)	71 (47,3)	108 (72,0)	42 (28,0)	134 (89,3)	16 (10,7)
	15 anos	35 (54,7)	29 (45,3)	50 (78,1)	14 (21,9)	60 (93,8)	4 (6,2)
	Valor de p (χ^2^)*	0,005		0,059		0,084	

* teste χ^2^.

## DISCUSSÃO

A VPI é o comportamento que provoca dano físico, sexual ou psicológico a uma mulher, exercida por parceiros íntimos atuais ou anteriores.^(3)^ Considerando tal premissa, os resultados do presente estudo indicaram que a escolaridade da mulher e a do parceiro foram fatores protetivos para evitar a ocorrência dos diferentes tipos de violência, principalmente quando o nível de escolaridade de ambos os parceiros é maior.^(15-19)^

Os achados demonstraram maior frequência de violência psicológica em mulheres separadas e divorciadas, indicando que a estabilidade em um relacionamento poderia favorecer a menor ocorrência desse tipo de evento.^(6,20)^

A totalidade das participantes deste estudo indicou ter sofrido algum tipo de VPI. Uma explicação possível para tal ocorrência poderia ser uma conversão de fatores, como a soma de diferentes participantes que tiveram distintos tipos de violência vivenciada, questões culturais de submissão do feminino ao masculino^(3)^ e o local de pesquisa ser uma Unidade Básica de Saúde do SUS.^(10)^

A forma de violência com maior prevalência foi a psicológica, o que confirma a hipótese inicial do estudo, seguida da física e da sexual, resultados similares aos da literatura.^(17,20,21)^ Ainda que a violência psicológica não deixe marcas físicas, ela é igualmente negativa e prejudicial para a saúde da mulher.^(5,22)^

Dentre os tipos de violência psicológica, o insulto e a intimidação são compreendidos como as formas de violência que mais causam prejuízo emocional e diminuição da autoestima, pois intencionam controlar as ações, os comportamentos, as crenças e as tomadas de decisões.^(8,9,23)^

A frequência da ocorrência da violência física (30%), nesta pesquisa, permaneceu conforme os índices de outros países, que variam de 13%, no Japão, a 61%, no Peru. O comportamento violento dos parceiros poderia ser associado ao sentimento de dominação e de controle sobre a companheira,^(24)^ o que tende a justificar o achado sobre a recorrência no episódio de violência.

A violência sexual identificada (12,3%)é maior do que em estudos realizados no Nepal^(24)^ e no México,^(2)^ mas menor do que um estudo com mulheres tailandesas.^(25)^ A violência sexual raramente ocorre de forma isolada, sendo fator de risco para vários agravos à saúde da mulher, com repercussões duradouras em sua vida.^(3)^

Dentre os tipos de violência sexual mais frequentes, foi identificada a relação sexual forçada. Outros estudos^(3,6)^ indicaram a ocorrência de relação sexual para tentar manter a própria integridade. Tal situação, poderia ser explicada pela discrepância na compreensão do que é violência sexual para a mulher e para o homem, pois ainda existe a ideia social de que a mulher deve realizar o ato sexual somente pela obrigação de satisfazer o desejo masculino.^(26)^

Ao serem indagadas sobre o que as participantes consideravam fatores predisponentes ao ato de VPI, a maioria indicou o uso de álcool pelo parceiro, conforme outros estudos^(16,24)^ que ressaltam que o álcool não deve ser usado pelas autoridades de poder como explicação para condutas violentas, mas como agravador delas. Ainda, alguns estudos^(2,3,16)^ indicam que o hábito do abuso de álcool pode aumentar o risco em até quatro vezes da ocorrência de violência sexual, dez vezes para violência física e cinco vezes para a violência psicológica.

Com relação às atitudes de gênero, foi possível identificar que, apesar de não haver motivo plausível que justifique um ato de violência,^(3)^ parcela das participantes considera possíveis explicações para a violência sofrida, como a infidelidade e a desobediência femininas. Algo semelhante foi encontrado no estudo da OMS realizado em áreas urbanas do Brasil, Japão, Namíbia e Sérvia e Montenegro,^(3)^ e também pode ser observado em Bangladesh, Etiópia, Peru, Samoa, Egito^(1)^ e Tailândia.^(25)^

A consideração de uma possível justificativa para a violência sofrida denota atitudes de submissão e conformismo da mulher em relação ao parceiro, confirmando a segunda hipótese do estudo, visto que a submissão contínua, ao longo dos anos, diminui a autoestima e a capacidade de pensar e a de reagir.^(27)^ Assim, a esperança de término da situação de violência cede espaço ao conformismo.^(3,28)^

O conformismo tende a banalizar a violência, que passa a ser vista como algo natural. Isso faz com que algumas mulheres assumam posturas passivas, como se essa fosse a resposta mais adequada para uma possível mudança comportamental do parceiro.^(27)^

As atitudes de submissão e subserviência, como as aqui descritas, podem estar associadas a uma criação rígida, baseada na subordinação ao sexo masculino, o que a Convenção Interamericana para Prevenir, Punir e Erradicar a Violência Contra a Mulher, em seu Art. 6º, busca desconstruir, ao afirmar que toda mulher tem o direito de não ser discriminada e de ser valorizada e educada, de forma a garantir igualdade entre gêneros.^(7)^

O presente estudo tem limitações, como ter sido realizado em um único local e com amostra que se forma por conveniência. Esse fato pode gerar resultados com limitações em sua capacidade de generalização. Ainda, a divisão da população em grupos menores, como por tipo de violência, atitude de gênero ou renda familiar, originou pequenas subamostras, que impossibilitaram uma maior exploração dos dados pela análise estatística.

Apesar das limitações, este trabalho foi capaz de apresentar a vivência de diferentes tipos de violência por suas participantes, identificando ocorrências e características que permitem um melhor manejo da população estudada, a fim de reduzir a VPI sofrida por elas.

Finalmente, os achados indicam a necessidade de promover uma educação que vise à igualdade de gênero e à maior autoestima nas mulheres, inibindo a prática da VPI. Intervenções de controle da violência devem ser voltadas a todos os envolvidos, e não somente às vítimas.

## CONCLUSÃO

Esta pesquisa descreveu a violência física, psicológica e sexual cometida pelo parceiro íntimo contra mulheres atendidas no Sistema Único de Saúde em uma cidade do interior do nordeste brasileiro. A maior prevalência de violência encontrada foi a do tipo psicológica, seguida da física e da sexual. A escolaridade das mulheres e dos parceiros mostrou-se como fator protetivo para as diferentes formas de violência. O abuso de álcool foi reconhecido como fator predisponente de atos violentos praticados pelo parceiro. Com relação à atitude de gênero, o aspecto que mais chamou atenção foi a identificação de mulheres que consideram haver explicações que justificariam a violência sofrida, refletindo a cultura de gênero vivida pelas participantes.
